# *Trans*-Chalcone Plus Baicalein Synergistically Reduce Intracellular Amyloid Beta (Aβ_42_) and Protect from Aβ_42_ Induced Oxidative Damage in Yeast Models of Alzheimer’s Disease

**DOI:** 10.3390/ijms22179456

**Published:** 2021-08-31

**Authors:** Sudip Dhakal, Paul A. Ramsland, Benu Adhikari, Ian Macreadie

**Affiliations:** 1 School of Science, RMIT University, Bundoora, VIC 3083, Australia; sudip.dhakal@rmit.edu.au (S.D.); paul.ramsland@rmit.edu.au (P.A.R.); benu.adhikari@rmit.edu.au (B.A.); 2 Department of Immunology, Monash University, Melbourne, VIC 3004, Australia; 3 Department of Surgery, Austin Health, University of Melbourne, Heidelberg, VIC 3084, Australia

**Keywords:** bioactive compounds, baicalein, *trans*-chalcone, amyloid beta, Alzheimer’s disease, yeast

## Abstract

Finding an effective therapeutic to prevent or cure AD has been difficult due to the complexity of the brain and limited experimental models. This study utilized unmodified and genetically modified *Saccharomyces cerevisiae* as model organisms to find potential natural bioactive compounds capable of reducing intracellular amyloid beta 42 (Aβ_42_) and associated oxidative damage. Eleven natural bioactive compounds including mangiferin, quercetin, rutin, resveratrol, epigallocatechin gallate (EGCG), urolithin A, oleuropein, rosmarinic acid, salvianolic acid B, baicalein and *trans*-chalcone were screened for their ability to reduce intracellular green fluorescent protein tagged Aβ_42_ (GFP-Aβ_42_) levels. The two most effective compounds from the screens were combined in varying concentrations of each to study the combined capacity to reduce GFP-Aβ_42_. The most effective combinations were examined for their effect on growth rate, turnover of native Aβ_42_ and reactive oxygen species (ROS). The bioactive compounds except mangiferin and urolithin A significantly reduced intracellular GFP-Aβ_42_ levels. Baicalein and *trans*-chalcone were the most effective compounds among those that were screened. The combination of baicalein and *trans*-chalcone synergistically reduced GFP-Aβ_42_ levels. A combination of 15 μM *trans*-chalcone and 8 μM baicalein was found to be the most synergistic combination. The combination of the two compounds significantly reduced ROS and Aβ_42_ levels in yeast cells expressing native Aβ_42_ without affecting growth of the cells. These findings suggest that the combination of baicalein and *trans*-chalcone could be a promising multifactorial therapeutic strategy to cure or prevent AD. However, further studies are recommended to look for similar cytoprotective activity in humans and to find an optimal dosage.

## 1. Introduction

Alzheimer’s Disease (AD) is a progressive neurodegenerative disease. It is a major cause of death in the elderly due to dementia, with the important pathological hallmarks in the brain being loss of proteostasis, increased oxidative damage, amyloid plaques, neurofibrillary tau tangles inside neurons, altered biometal distribution, imbalance of neurotransmitters, lipid dyshomeostasis, neuronal death, genomic instability, cognitive impairment and loss of synapses [1]. Despite the huge effort to understand AD pathology and its cause, assigning a specific cause to AD’s multifactorial manifestations has been very difficult. Similarly, targeting one cause for treatment of the disease is not sufficient, which could be one important reason behind the limitation of current therapeutics [2]. Another point for consideration is that as AD progresses, it causes irreversible damage to neuronal cells which cannot be rejuvenated with current therapeutics [3]. The early slow progression of AD accelerates in later stages and becomes more severe as individuals grow older. Hence, a sustainable approach to prevention seems the best way forward.

Various phytochemicals have been investigated to find a cure or a preventative for AD [1,4]. Dietary polyphenols are a specific class of bioactive compounds that have gained great interest during recent years due to their multifactorial benefits [5]. Most polyphenols belong to several classes including stilbenes, flavonoids, coumarins, tannins, phenolics, lignans and lignin [6,7]. Regardless of the structure and classification of these polyphenols, polyphenolic compounds from each of these classes have been found to have significant positive effect in cellular health. Even the precursors of these polyphenols like chalcones have been found to have a multifactorial effect, and they can be modified easily possibly leading to chalcone-based novel synthetic drugs [8]. The most important properties of these compounds relevant to AD include their effects on antioxidant activity, cell cycle regulation, the proteostasis network, longevity, lipid homeostasis, biometal homeostasis, anti-inflammatory activity, anti-amyloidogenic activity, acetylcholine esterase/butyrylcholine esterase inhibition, cognition and synapses.

Considering such multifactorial effects, this study investigated the effectiveness of selected bioactive compounds with potential cytoprotective effects against AD. The compounds tested in this study were mangiferin, oleuropein, urolithin A, resveratrol, epigallocatechin gallate (EGCG), rosmarinic acid, salvianolic acid B, quercetin, rutin, baicalein and *trans*-chalcone. Most of these compounds are previously shown to have antioxidant, in vitro anti-amyloidogenic, anti-inflammatory and autophagy induction properties. These effects are regarded as excellent strategies to treat or prevent AD [1]. Furthermore, some of the selected compounds have unique abilities to chelate metals such as iron, reduce biometal cytotoxicity, induce lysosomal biogenesis, recover mitochondrial health, and provide other beneficial aspects which are highly relevant to AD pathology. Not all the compounds have the same anti-AD effect, and how they compare with each other in terms of their effectiveness and their combination abilities remains elusive. Hence, it is paramount to study their abilities when used together.

Unmodified and genetically modified *Saccharomyces cerevisiae* constitutively expressing intracellular GFP-Aβ_42_ and native Aβ_42_ were employed to examine the effect of bioactive compounds and their combinations. In previous studies, the GFP-Aβ_42_, observed as punctate patches, was only found expressed in the old cells, while the younger cells lacked green fluorescence, providing clues on their capacity to clear the fusion protein effectively [9,10]. This phenomenon aligns well with human ageing and advocates that the yeast ageing can be an excellent tool to understand eukaryotic ageing [10]. Further elaboration on how yeast provides an excellent platform for studying AD is reviewed elsewhere [11,12,13]. In another study, differential gene expression analysis between GFP-Aβ_42_ producing yeast compared with GFP producing yeast showed high expression of *AHP1* in GFP-Aβ_42_ producing cells [14]. *AHP1* encodes an allyl hydroperoxidase which is induced by Aβ_42_ to provide protection against the Aβ_42_. The human equivalent of this peroxidase is periredoxin 5 (Prx5) [15]. Following this observation, Δ*ahp1* deletant cells were evaluated to study its effect on GFP-Aβ_42_ expression. It was found that approximately 20–30% cells expressed GFP-Aβ_42_ [16]. In this study, such yeast cells were used as a model for studying the turnover of GFP-Aβ_42_ to screen the bioactive compounds that can reduce the levels of GFP-Aβ_42_. The two compounds identified as most effective during screening were then evaluated for their combined abilities to reduce GFP-Aβ_42_ and as well as Aβ_42_-induced ROS. Overall, the study demonstrates relative ease of screening bioactive compounds in combination to increase their beneficial effects, further demonstrating the utility of yeast models of AD as powerful tools to perform such studies.

## 2. Results

### 2.1. Baicalein and Trans-Chalcone Were the Most Effective Compounds to Reduce Green Fluorescent Cells

**Figure 1:**
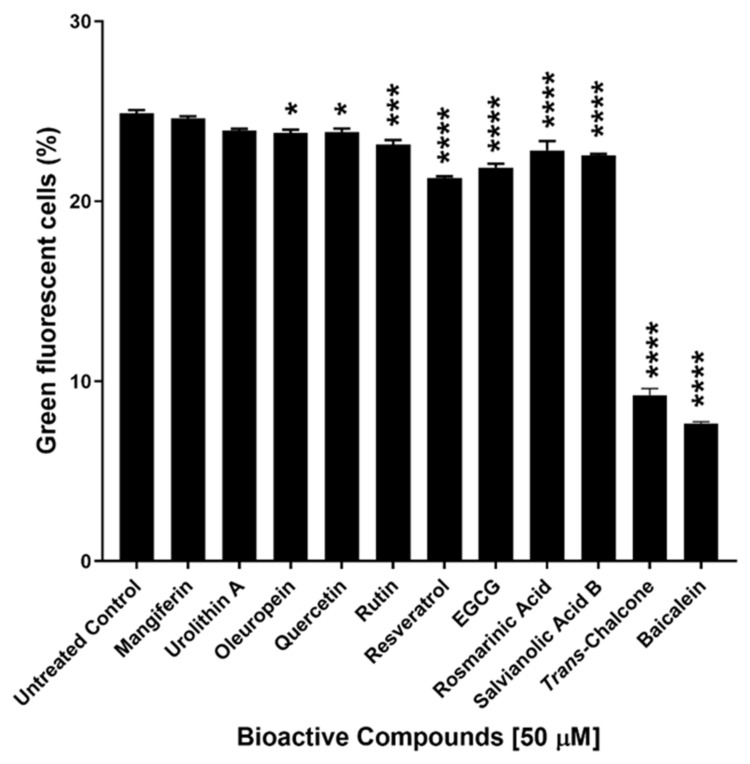
Screening of naturally occurring bioactive compounds by estimating their effects on green fluorescent cells in the population of BY4743 Δ*ahp1* [p416.GPD.GFPAβ_42_] transformants. Statistically significant values obtained from one-way ANOVA with reference to untreated control cells are represented by asterisks (* *p* < 0.05, *** *p* < 0.001 and **** *p* < 0.0001).

In this study, eleven naturally occurring bioactive compounds including mangiferin, urolithin A, oleuropein, quercetin, rutin, resveratrol, epigallocatechin gallate (EGCG), rosmarinic acid, salvianolic acid B, baicalein and *trans*-chalcone were selected (due to their known protective effect in cell health) and evaluated for their effect on GFP-Aβ_42_ expression in yeast cells. Nine out of eleven of these compounds significantly reduced the levels of GFP-Aβ_42_. Baicalein and *trans*-chalcone were found to be the most effective compounds enabling ~60–70% reduction in cells with GFP-Aβ_42_ (Figure 1).

### 2.2. Combination of Baicalein and Trans-Chalcone Significantly Reduced Levels of GFP-Aβ_42_ as Compared to Single Compound Treatments

**Figure 2:**
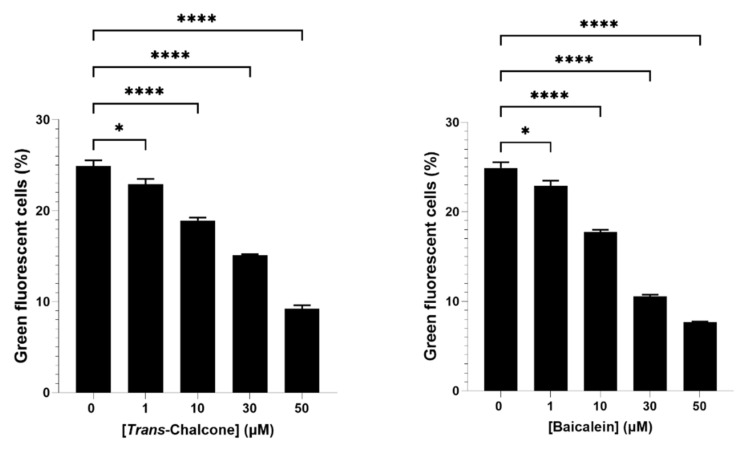
Population of BY4743 Δ*ahp1* [p416.GPD.GFPAβ_42_] with green fluorescence following exposure to *trans*-chalcone (left panel) and baicalein (right panel). Statistically significant values obtained from one-way ANOVA test in reference to untreated control are represented by asterisks (* *p* < 0.05 and **** *p* < 0.0001).

Based on the results from screening of bioactive compounds (Figure 1), baicalein and *trans*-chalcone were selected for further evaluation on how their combination affected GFP-Aβ_42_ levels in yeast models. Prior to that, a dose response assay on GFP-Aβ_42_ expression was also performed to check any underlying dose effect of the compounds (Figure 2). Both the baicalein and *trans*-chalcone reduced the levels of GFP-Aβ_42_ in a dose-dependent manner with slightly higher reduction demonstrated by baicalein.

**Figure 3:**
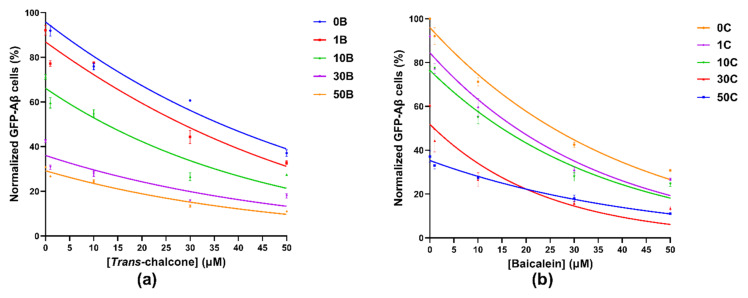
Gaussian curves obtained by plotting the normalized GFP-Aβ_42_ positive cells in percentages when the BY4743 Δ*ahp1* [p416.GPD.GFPAβ_42_] cells were treated with (**a**) combinations of 0, 1, 10, 30 and 50 µM *trans*-chalcone with constant concentrations of baicalein at 0, 1, 10, 30 and 50 µM represented by curves 0B, 1B, 10B, 30B and 50B, respectively, and (**b**) combinations of 0, 1, 10, 30 and 50 µM baicalein with constant concentrations of *trans*-chalcone at 0, 1, 10, 30 and 50 µM represented by curves 0C, 1C, 10C, 30C and 50C, respectively. The total GFP-Aβ_42_ producing cells in the untreated control were normalized to 100% and used for comparison with the treated populations.

To investigate the combination effect of baicalein and *trans*-chalcone, yeast cells producing GFP-Aβ_42_ were treated with combinations of varying concentrations of baicalein and *trans*-chalcone. The combination of the two chemicals showed enhanced ability to reduce GFP-Aβ_42_ as compared to single compound treatments (Figure 3). The treatment with the combination of the compounds showed a significant combination effect in almost all the treatments with some exceptions. The findings suggested that an equal amount of the two compounds showed significant synergy compared to other combinations evident by highest outcome in cells treated with a combination of 50 µM each. The combination of 50 μM baicalein and 30 μM *trans*-chalcone was the second best in terms of reduction of GFP-Aβ_42_. Similarly, the combination treatments 30 μM *trans*-chalcone with 30 μM baicalein and 50 μM *trans*-chalcone with 30 μM baicalein were also found to have similar effects but with slightly lower reduction in all other combinations.

Data obtained after treating the GFP-Aβ_42_ transformants with combinations of baicalein and *trans*-chalcone were represented by Gaussian curves (Figure 3), which shows that the combined effect of the two compounds in reducing the GFP-Aβ_42_ positive cell population was beneficial.

**Figure 4:**
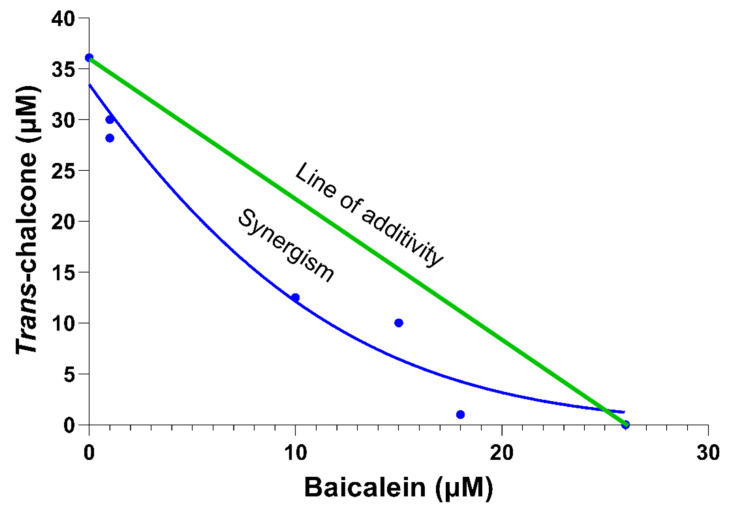
An isobologram created using the 50% inhibitory concentration (IC50) that reduced 50% of the GFP-Aβ_42_ positive cells after treating BY4743 Δ*ahp1* [p416.GPD.GFPAβ_42_] cells with baicalein, *trans*-chalcone and their combinations. The *X*-axis represents concentration of baicalein; *Y*-axis represents the concentration of *trans*-chalcone; green line is the theoretical line of additivity; blue dots are the observed IC50 values obtained after treating the cells with the combinations; and the blue curved line is a Gaussian curve showing the combination of baicalein and *trans*-chalcone was synergistic in reducing GFP-Aβ_42_.

Using the curves from Figure 3, the 50% inhibitory dose (IC50) for the combinations of baicalein and *trans*-chalcone were determined. An isobologram was created to assess underlying relationship between the two compounds (Figure 4). From Figure 4, it can be deduced that the baicalein and *trans*-chalcone acted in synergy, shown by the increased effect when used in combination [17].

### 2.3. The Most Effective Combination of Baicalein and Trans-Chalcone to Show Synergy in Reducing GFP-Aβ_42_ Was Not Growth Inhibitory

**Figure 5:**
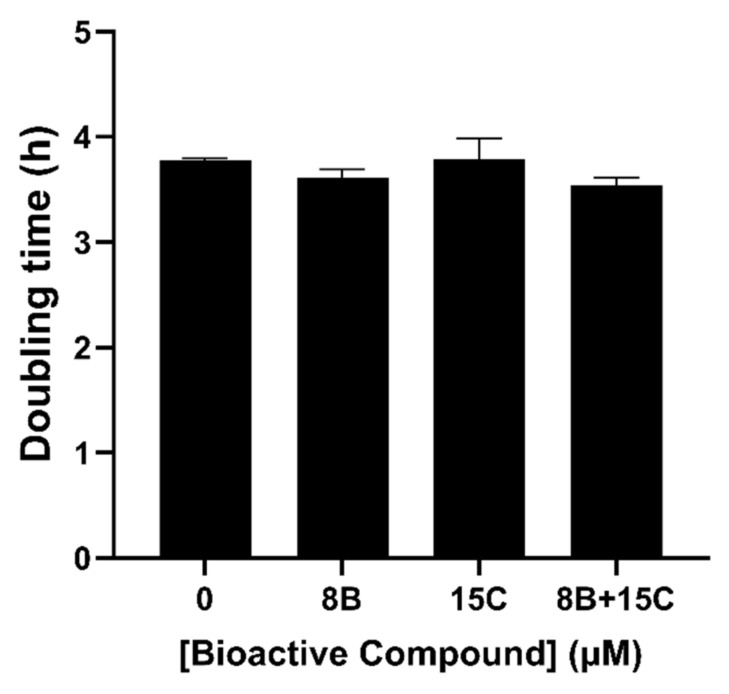
Doubling time of BY4743 [pYEX.Aβ] after exposure to 8 μM baicalein (8B), 15 µM *trans*-chalcone (15C) and their combination (8B + 15C) in minimal media supplemented with the auxotrophic requirements of the strain.

From the isobologram (Figure 4), the synergistic combination with lowest concentration was expected at 15 μM *trans*-chalcone and 8 μM baicalein. Hence, the combination of 8 μM baicalein and 15 µM *trans*-chalcone was evaluated for potential growth inhibition effect by measuring the optical density at 600 nm (OD_600_) wavelength at different time points of freshly grown suspension culture of native Aβ_42_ transformants (Figure 5). A linear region in the growth curve was identified, and the OD_600_ value was converted to population doubling time. The results obtained showed that there was no difference in the population doubling time of the cells treated with the combination of baicalein and *trans*-chalcone.

### 2.4. 8 μM Baicalein Plus 15 μM Trans-Chalcone Does Not Inhibit Growth, Reduces GFP-Aβ_42_ and Rescues Cells from Aβ_42_-Induced ROS

**Figure 6:**
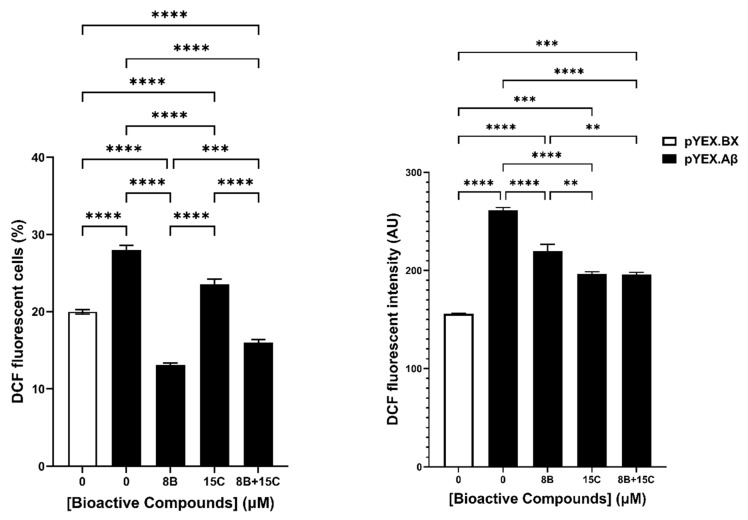
Effect on intracellular reactive oxygen species (ROS) in BY4743 [pYEX.Aβ] cells treated with 8 µM baicalein (8B), 15 µM *trans*-chalcone (15C) and their combination (8B + 15C). pYEX.BX represents data obtained from BY4743 [pYEX.BX] empty vector control and pYEX.Aβ represents data obtained from BY4743 [pYEX.Aβ] transformants. Left panel shows the change in percentage of dichlorofluorescein (DCF) fluorescent cells and right panel shows the median DCF fluorescence intensity differences between the treatments. Statistically significant values obtained by one-way ANOVA analysis are represented by asterisks (** *p* < 0.01, *** *p* < 0.001 and **** *p* < 0.0001).

The combination 15 μM *trans*-chalcone and 8 μM baicalein was tested to evaluate its effect on the intracellular reactive oxygen species in BY4743 [pYEX.Aβ] cells (Figure 6). The measurement of ROS was performed by using 2′,7′-dichlorodihydrofluorescein diacetate (H_2_DCF-DA) staining in two different ways. At first, the percentage of cells that are ROS positive were evaluated. The results obtained depicted significant reduction in ROS positive cells with the combination as well as with the individual compounds. Likewise, the evaluation of median fluorescence intensity representing single cell ROS levels showed similar pattern of decrease in DCF intensity. In summary, it was found that the combination of 8 µM baicalein and 15 µM *trans*-chalcone reduced ROS induced by Aβ_42_ at a significant level, but the combination did not reduce ROS in a synergistic manner.

### 2.5. 15 μM Trans-Chalcone and 8 μM Baicalein Significantly Reduced Aβ_42_ Levels

**Figure 7:**
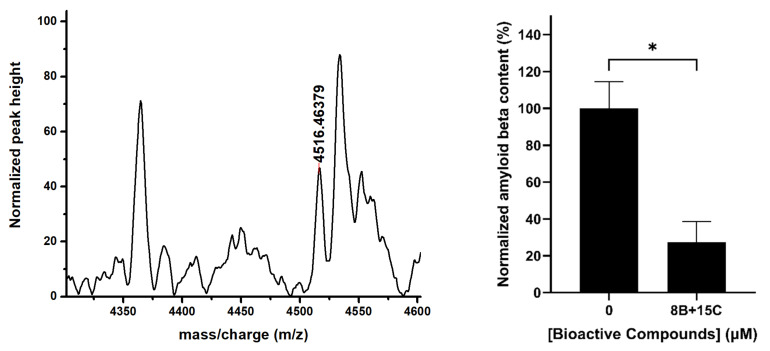
Quantification of intracellular Aβ_42_ in BY4743 [pYEX.Aβ] cells treated with *trans*-chalcone and baicalein. Left panel showing mass spectrum of cell lysate protein peaks near the expected molecular weight (4516 Da) of Aβ_42_ obtained from MALDI-TOF MS. Right panel shows levels of Aβ_42_ protein in BY4743 [pYEX.Aβ] cells treated with the combination of 8 μM baicalein and 15 μM *trans*-chalcone (8B + 15C). Statistically significant values obtained from unpaired t-test for difference in mean are represented by asterisks (* *p* < 0.05).

The combination of 15 μM *trans*-chalcone and 8 μM baicalein was also evaluated for its ability to reduce Aβ_42_ toxic protein in BY4743 [pYEX.Aβ] transformants in one generation time (Figure 7). The levels of Aβ_42_ were quantified using MALDI-TOF mass spectrometry in lysates of treated cells and compared with the untreated controls using a standard curve. The Aβ_42_ protein content was found to be significantly reduced (~60–70% reduction) in the cells treated with the combination of 8 µM baicalein and 15 µM *trans*-chalcone with reference to the untreated control supporting previous findings of the benefits of using the combination.

## 3. Discussion

Oxidative damage and aggregation of proteins are common manifestations of aging and are highly increased during AD [18,19]. Increasing evidence supports loss of proteostasis as the major pathological hallmark of AD, evident by accumulation of amyloid plaques and tau neurofibrillary tangles in the brain [12]. The toxicity of Aβ_42_ peptide and ability to enhance intracellular ROS, damage mitochondria, cause stress and formation of amyloid plaques restricting brain function has been considered a major cause of AD [9,20,21]. Recent studies supporting increased amyloid content in AD brains and the fact that diseased neurons release exosomes with oligomeric forms of Aβ_42_ from neurons which spread to the neighbouring neurons has been found to be crucial for AD progression [22,23]. Considering these aspects of Aβ_42_ and its toxicity towards cellular health, this study utilised yeast models for studying Aβ_42_ turnover and screening of bioactive compounds to cure or prevent AD.

Naturally occurring dietary polyphenols consist of aromatic phenolic groups with various hydroxylation patterns, and they can readily contribute their hydrogen ions to the oxidized species within cells and interact with aggregation-prone proteins [24,25]. These compounds, present in various foods including fruits and vegetables, have been reported to promote healthy aging [26]. Their multifactorial effect in AD pathology is promising, leading to several clinical trials on AD [1,27]. Such great attention to polyphenols could also be due to their amphiphilic nature and their ability to cross the blood–brain barrier [28]. Considering these beneficial effects of polyphenols and their precursors, eleven selected bioactive compounds with potential to cure/prevent AD were chosen in this study. Although there are thousands of bioactive compounds available in our diet and medicinal plants, these eleven compounds were chosen based on their multifactorial anti-AD properties shown in previous studies. However, some compounds with beneficial effects against AD such as curcumin were not selected for this study because of their natural green fluorescence properties which interferes with the evaluation of the green fluorescence used in this study [29].

At first, the compounds were screened for their ability to reduce the proportion of cells with GFP-Aβ_42_ in the yeast population to find the compounds with potential benefits. Old cells do not normally remove Aβ_42_ or GFP-Aβ_42_; however, some bioactive compounds can support these older cells to clear these proteins. The results obtained showed that approximately 25% of the BY4743 Δ*ahp1* deletant cells were GFP-Aβ_42_ positive. While the expression of the fusion protein GFP-Aβ_42_ was controlled under a constitutive promoter and was expected to be expressed at constant rate in all cell types, only one fourth of the population showed GFP-Aβ_42_ positivity. In fact, it was the grandmothers and older yeast cells which account for nearly 25% of the population that produce GFP-Aβ_42_ due to loss of proteostasis in older cells [10]. Another point that needs to be noted is that the yeast strain used in this study is devoid of the alkyl hydroperoxide reductase (*AHP1*) gene, a homolog of human peroxiredoxin 5 (*PRX5*), which expresses a protein that rescues cells from lipid peroxidation and oxidative damage induced by Aβ_42_ [15]. In addition, the *AHP1* deletant strains have been previously shown to have 20–30% GFP-Aβ_42_ positive cells in a freshly grown population of yeast cells suggesting potential role of *AHP1* in proteostasis maintenance [13]. Among the compounds tested, all the compounds except mangiferin and urolithin A significantly reduced the proportion of cells with GFP-Aβ_42_ with the highest reduction occurring with baicalein and *trans*-chalcone treatments. Both the compounds reduced GFP-Aβ_42_ in a dose-dependent manner with IC50 values of approximately 26 µM and 36 µM. The combination of baicalein and *trans*-chalcone was tested for their ability to reduce GFP-Aβ_42_ in combination based on the highest effect observed in the first screening.

Although previous studies have shown beneficial individual effects of baicalein and *trans*-chalcone in AD models [1,8,30], this is the first report to show their increased effect in combination. Baicalein and *trans*-chalcone showed a synergistic effect in reducing the intracellular GFP-Aβ_42_. A combination of 8 µM baicalein and 15 µM *trans*-chalcone was the combination with lowest amount of two compounds with synergistic effect in reducing GFP-Aβ_42_. The combination 8 µM baicalein and 15 µM *trans*-chalcone was not growth inhibitory. In fact, the combination also reduced cellular ROS induced by Aβ_42_ and approximately 60–70% of the native Aβ_42_ levels inside the yeast cells as evaluated by mass spectrometry. These findings suggest that the combination of baicalein and *trans*-chalcone could be a novel strategy to prevent the AD by decreasing the levels of Aβ_42_. However, the concentration of the two compounds to be used in humans could be drastically different due to factors such as bioavailability, metabolism and intraneuronal environment.

Baicalein, a flavonoid, has been reported to scavenge ROS and activate transcriptional factor NF-E2-related factor 2 (Nrf2) in human neuroblastoma cells (SH-SY5Y cells) [31]. The activated Nrf2 transcription factor induces expression of an array of antioxidant response element-dependent genes relieving the oxidative stress [32]. Apart from the abovementioned antioxidant effect, baicalein was reported to chelate iron and reduce iron mediated Fenton chemistry [33]. This indicates its potential to be used as an AD treatment strategy to reduce or prevent the iron overloading, ferroptosis and formation of lipofuscin characteristic in the AD brain microenvironment. In in vitro studies, baicalein was found to have anti-amyloid activity depicted by disaggregation of amyloid aggregates and reduction in the formation of oligomeric and fibrils forms of Aβ_42_ [34]. Baicalein treatment also induced alpha-secretase activity and reduced Aβ_42_ formation in Chinese hamster ovary (CHO) cells that were genetically engineered to produce amyloid precursor protein (CHO/APPwt) [35]. In addition, it also possesses anti-inflammatory effects by inhibiting tumour necrosis factor α (TNFα)-induced nuclear factor kappa B (NF*k*B) activation and subsequent expression of pro-inflammatory mediators [36]. Additionally, baicalein has also been reported to reduce the formation of α-synuclein aggregates and to disaggregate its pre-formed fibrils [37]. Recent studies revealing reduced tau fibrillization and solubilization of the tau proteins after baicalein treatment could be highly beneficial in improving cognitive impairment due to the formation of tau neurofibrillary tangles in AD patients [38]. Such demonstration of disaggregation and reduction of toxic protein aggregates associated with disrupted proteostasis network in AD models hints towards its potential importance in maintaining the cellular proteostasis network. Furthermore, baicalein has been found to recuperate the mitochondrial health and improve biogenesis of mitochondria in rotenone-induced Parkinson’s Disease in rat models of PD [39]. Importantly, baicalein also restored lysosomal acidity by enhancing vacuolar ATPase (v-ATPase) assembly in hepatic cells of mice fed with high fat diets [40]. The v-ATPase is the lysosomal transmembrane hydrogen ion pump that maintains the acidity of the lysosomes and endosomes which is essential for activity of the resident proteases such as cathepsins [12]. Previous studies indicate biological ageing of cells are accompanied by loss of lysosomal acidity and stands as one of the causes for loss of proteostasis in ageing. In addition, baicalein is also demonstrated as the most potent flavonoid to inhibit acetylcholine esterase (AChE) in in vitro studies [41]. Hence, the benefits provided by baicalein have a multifactorial nature that can help recover mitochondrial health, reduce oxidative damage, enhance synaptic plasticity, clear aggregated toxic proteins and defective autophagic vesicles, improve cognition and proteostasis, all of which are important to combat AD. However, the growth inhibitory effect of baicalein needs to be addressed, possibly by addition of *trans*-chalcone or similar bioactive compounds in the treatment regimen.

Similarly, *trans*-chalcones, a precursor for flavonoids, are also reported to have beneficial effects in disease models for AD. Male Wistar rats induced for formation of amyloid plaques treated with the *trans*-chalcone showed reduced accumulation of amyloid plaques in their brain [42]. *Trans*-chalcones are shown to have hepatoprotective, anti-microbial, anti-diabetic, anti-cancer, anti-obesity, hypolipidemic, anti-inflammatory, cytoprotective and antioxidant properties that are beneficial to a wide range of chronic diseases with multifactorial manifestation including AD [43]. Additionally, *trans*-chalcones are bioactive compounds that can be converted by chemical modifications to other derivatives to diversify and possibly increase their effects [8]. Recent studies on *trans*-chalcone derivatives have been of great interest in most of the chronic diseases of humans due to their small molecular size, convenient cost-effective synthesis, and ease to modify their lipophilicity [44]. In a previous study, *trans*-chalcone is also reported to induce apoptosis and growth arrest in cancer cell lines [45]. However, the effect of bioactive compounds is largely dependent on various factors like cellular environment, dosage, and bioavailability. On the other hand, growth arrest and proliferation inhibition due to *trans*-chalcone treatment in terminally differentiated neurons could also be beneficial for their survival and lifespan [46]. To overcome the limitations of treatment with individual therapy, it is possible to have significant benefits by addition of bioactive compounds like baicalein. This study sets an example for the possibilities of exploring such combinations and for a need for further research.

From the findings of this study, it can be concluded that the combination of the baicalein and *trans*-chalcone is likely to have increased benefits possibly without causing cellular growth defects in AD patients. We suggest the combination of baicalein and *trans*-chalcone for future studies to develop a therapeutic or preventative strategy against AD.

This study is yet another illustration of using yeast AD models to rapidly find therapeutic or preventative agents against AD. There are several other studies possible in yeast models discerning the molecular mechanisms on how these compounds carry out their beneficial effects. The natural availability and reduced toxicity of such bioactive compounds has been a great motivation for further investigation. The combination of the bioactive compounds with other drugs or bioactive compounds could pave the way forward to target the causes of AD in a multifactorial manner. However, numerous challenges are to be addressed in humans due to the complex cellular environment and several layers of metabolic barriers for the bioactive compounds to reach the brain. Future research on how to increase their bioavailability in the body or brain and the possibilities of new combinations with the current findings could help find efficient therapeutic strategies against AD.

## 4. Materials and Methods

### 4.1. Strains of Yeast and Culture Media

In this study, the *Saccharomyces cerevisiae* BY4743 *Δahp1* (*MATa/α his3Δ1/his3Δ1, LYS2/lys2Δ0 met15Δ0/MET15 ura3Δ0/ura3Δ0 leu2Δ0/leu2Δ0*) were transformed with plasmids p416GPD.GFPAβ_42_ and p416GPD.GFP; BY4743 (*MATa/α his3Δ1/his3Δ1, LYS2/lys2Δ0 met15Δ0/MET15 ura3Δ0/ura3Δ0 leu2Δ0/leu2Δ0*) with pYEX.BX and pYEX.Aβ as described previously [15,47]. The strains were named as *S. cerevisiae* Strain [plasmid name] accordingly after transformation with the respective plasmids.

Minimal media, comprising 0.67% yeast nitrogen base (YNB) (Sigma-Aldrich Pty Ltd., Sydney, Australia), 2% dextrose, plus the auxotrophic requirements of the yeast transformants, were used to grow cells as described previously [15,47,48].

### 4.2. Screening of Bioactive Compounds That Reduce Expression of GFP-Aβ_42_

The bioactive compounds were assessed for their anti-amyloid properties in two different ways. At first, the bioactive compounds were evaluated for their ability to change expression of GFP-Aβ_42_ in yeast transformants as described previously [16].

Freshly grown overnight culture of *S. cerevisiae* BY4743 Δ*ahp1* [p416.GPD.GFPAβ_42_] transformants were refreshed with freshly prepared minimal media for 2–3 h at 30 °C shaking at 200× *g*. The yeast transformants were then treated with a set of selected bioactive compounds purchased from Sigma-Aldrich Pty Ltd., Sydney, Australia including mangiferin, oleuropein, urolithin A, rutin, quercetin, resveratrol, epigallocatechin gallate (EGCG), rosmarinic acid, salvianolic acid B, baicalein and *trans*-chalcone at final concentration of 50 μM each for 2 h at 30 °C in 48-well tissue culture plates shaking at 100× *g*. Following the incubation, yeast cells were fixed with paraformaldehyde at final concentration of 3.7% for 3–5 min. The fixed cells were washed twice using phosphate buffered saline (PBS) and analysed using a BD LSRFortessa™ Flow Cytometer (BD Biosciences, Sydney, Australia). The cells were examined for green fluorescence using the excitation wavelength of 488 nm and emission at 530 nm.

### 4.3. Evaluation of Combination Effect of Two Most Effective Compounds for Their Ability to Reduce GFP-Aβ_42_ Levels

Considering results from screening, the two most effective compounds were selected and tested for their combination effect in reducing GFP-Aβ_42_ levels in yeast cells. In doing so, BY4743 [p416.GPD.GFP-Aβ] transformants were freshly grown overnight and refreshed for 2–3 h at 30 °C with shaking at 200× *g*. Standardized cultures (approximately 10^7^ cells) of yeast transformants were treated with varying final concentration of 0, 1, 10, 30 and 50 μM of the compounds and all possible combinations of the two compounds in the concentration range (0, 1, 10, 30, 50 μM for each compound) for 2 h at 30 °C with shaking at 100× *g*. After treating the cells, they were fixed and washed before analysis through flow cytometer for evaluation of green fluorescent cells as described in Section 4.2. The results obtained from the assay were analysed to determine the 50% inhibitory dose (IC50) that can reduce exactly half of the GFP-Aβ_42_ producing cells for all the combinations as well as the individual treatments. An isobologram was created using the obtained IC50 values, and the observed effect of the combination of the compounds were evaluated to determine if the observed combination effect was antagonistic, additive or synergistic [17].

### 4.4. Growth Inhibition Assay

Growth inhibition assays were performed to investigate if the observed reduction of GFP-Aβ_42_ in yeast cell was associated with growth inhibition due to treatment with the most effective combination of bioactive compounds. The compounds were evaluated for growth rate in freshly grown BY4743 [pYEX.Aβ] by measuring optical density of suspension culture at 600 nm wavelength (OD_600_) at different time points (0, 1.5, 3, 5, 20 and 24 h) of the incubation. The cells were treated with a combination of baicalein and *trans*-chalcone obtained from isobologram that is expected to have lowest concentration of each compound with excellent synergy. The obtained OD_600_ values were used to calculate growth rates in various treatment and data obtained were statistically analysed using GraphPad Prism 9 software (GraphPad Software, San Diego, CA, USA).

### 4.5. Measurement of ROS Using 2′,7′-Dichlorodihydrofluorescein Diacetate (H_2_DCFDA) Staining

The intracellular ROS produced in BY4743 [pYEX.Aβ] after the treatment with most effective combination of compounds was estimated with the previously described method using 2′,7′-dichlorodihydrofluorescein diacetate (H_2_DCFDA) stain purchased from Sigma-Aldrich Pty Ltd, Sydney, Australia [47]. The yeast cells were freshly grown overnight and refreshed for 2–3 h prior to treating them with the compound combination (15 μM *trans*-chalcone and 8 μM baicalein). The standardized cultures of fresh cells (~10^6^ cells) were then subjected to chemical treatment for 2 h at 30 °C with shaking at 100 rpm. After 1 h incubation with the chemicals, the suspension culture was added with H_2_DCFDA at the final concentration of 10 μg/mL for each reaction. The culture plate was wrapped with aluminum foil and incubated at 30 °C for 1 h with shaking at 100× *g*. Unstained and untreated native Aβ transformants along with untreated and unstained BY4743 [pYEX.BX] empty vector controls were analysed. A positive control empty vector transformant yeast suspension treated with 1% hydrogen peroxide was used to validate the test. After incubating cells for 2 h, the cells were washed with sterile water by centrifuging cells at 3000 rpm for 3 min. The washed cells were immediately added with fresh media and incubated for 1 h at 30 °C with shaking at 100 rpm. Following the incubation, the yeast cells were fixed at final concentration of 3.7% paraformaldehyde for 3–5 min. The fixed cells were washed twice with PBS before analysis for green fluorescence with a BD LSRFortessa™ Flow Cytometer using parameters similar to those in Section 4.2. Data obtained were analysed using FlowJo version 10.6.1 (FlowJo LLC., Ashland, OR, USA).

### 4.6. Determination of Aβ_42_ Protein Levels by MALDI-TOF MS

The Aβ_42_ protein content in cells treated with the most effective combination of the two bioactive compounds were performed using a mass spectrometry-based method used previously with slight modifications [46].

#### 4.6.1. Chemical Treatment of Yeast Cells and Extraction of Cell Lysates for Determination of Amyloid Beta Turnover

BY4743 [pYEX.Aβ] and BY4743 [pYEX.BX] were cultured overnight and freshened for 2–3 h with fresh minimal media at 30 °C with shaking at 200× *g*. Following the freshening of the yeast transformants in the liquid media supplemented with auxotrophic requirements essential for growth, BY47473 [pYEX.Aβ] were treated with the combination of 15 μM *trans*-chalcone and 8 μM baicalein for two hours at 30 °C with shaking at 200× *g*. The study was performed along with the untreated control pYEX.Aβ and pYEX.BX transformants. After two hours of incubation, the yeast cells were harvested by centrifugation at 3000× *g* for 3 min, and cells were lysed using 0.5 mm beads in a FastPrep-24™ Classic bead beating grinder and lysis system (MP Biomedicals LLC., Irvine, CA, USA) at high speed for 1 min for 6 cycles. To avoid the heating of the tubes, each cycle of homogenization was followed by 1-min rest period on ice. The lysate obtained was analysed for the level of Aβ_42_ using MALDI-TOF mass spectrometry (Bruker Pty Ltd., Preston, VIC, Australia).

#### 4.6.2. MALDI-TOF Analysis of the Aβ_42_ Levels in Yeast Cell Lysates

The protein content of each sample was quantified using Nanodrop (Thermo Fisher Scientific Australia Pty Ltd., Victoria, Australia), and the protein content in each sample was standardized. A set of standards of different concentrations (0.31, 0.63, 1.25, 2.5 and 5 μM) of Aβ_42_ was prepared with the lysate of BY4743 [pYEX.BX] as background to standardize the ion suppression in protein mix of lysate. The mass spectrum for each sample was collected and the data obtained was analysed to determine the standard curve of amount of Aβ_42_ versus peak height. To normalize the variation in the data acquisition, a constant amount of internal control (known molecular weight—3637 Da) was added in each sample spot before data acquisition. The MALDI-TOF MS analyser was calibrated using protein calibration standard prior to data acquisition. The mass spectrum obtained from standards and samples was analysed using OriginPro 2016 statistical software (Originlab Corporation, Northampton, MA, USA) for peak deconvolution and identification of the peak at a range of mass to charge ratio equivalent to molecular weight of Aβ_42_ (4515–4517 Da). The peak height data were used for comparing different samples and determination of standard curve. The peak height data obtained were converted to the amount of Aβ_42_ in each sample spot using the standard curve. The Aβ_42_ content in the sample lysates was spiked to a detectable range by addition of known constant amount of synthetic Aβ_42_. The difference in the Aβ_42_ between the lysate of native Aβ transformant and empty vector control was estimated to be the total Aβ_42_ present in native Aβ transformants. Similarly, the Aβ_42_ content in the lysates of the native Aβ transformants treated with the combination of *trans*-chalcone and baicalein were determined and compared with that of the untreated control native Aβ transformant.

## 5. Conclusions

In summary, this study signifies the importance of yeast AD models to identify drug combinations that can act against Aβ_42_. In addition, baicalein and *trans*-chalcone have been identified to possess the most effective activity in comparison to other bioactive compounds tested in this study. The combination of baicalein and *trans*-chalcone was more beneficial than individual treatments indicating enormous potential of the combination to be used as safe and effective therapeutic strategy to cure or prevent AD.

In the future, it is recommended that determining the molecular mechanism behind enhanced potential of baicalein and *trans*-chalcone, particularly which cytoprotective proteins are activated and which are switched off, could provide further insight in determining effective therapeutic strategy against amyloid build up in AD patients. Further research in human AD patients could provide evidence for the positive benefits of these bioactive compounds and could lead to production of excellent nutraceutical products. In addition, investigation of other bioactive compounds not included in this study is also recommended to find out if they can be supplemented in the regimen to gain increased multifactorial benefits against AD.

## Data Availability

Not applicable.
